# Exploration of a Novel Prognostic Nomogram and Diagnostic Biomarkers Based on the Activity Variations of Hallmark Gene Sets in Hepatocellular Carcinoma

**DOI:** 10.3389/fonc.2022.830362

**Published:** 2022-03-10

**Authors:** Xiongdong Zhong, Xianchang Yu, Hao Chang

**Affiliations:** ^1^ Department of Cardiothoracic Surgery, Zhuhai People’s Hospital (Zhuhai Hospital Affiliated with Jinan University), Zhuhai, China; ^2^ Department of Protein Modification and Cancer Research, Hanyu Biomed Center Beijing, Beijing, China

**Keywords:** prognosis, diagnosis, GSVA, nomogram, hepatocellular carcinoma

## Abstract

**Background:**

The initiation and progression of tumors were due to variations of gene sets rather than individual genes. This study aimed to identify novel biomarkers based on gene set variation analysis (GSVA) in hepatocellular carcinoma.

**Methods:**

The activities of 50 hallmark pathways were scored in three microarray datasets with paired samples with GSVA, and differential analysis was performed with the limma R package. Unsupervised clustering was conducted to determine subtypes with the ConsensusClusterPlus R package in the TCGA-LIHC (*n* = 329) and LIRI-JP (*n* = 232) cohorts. Differentially expressed genes among subtypes were identified as initial variables. Then, we used TCGA-LIHC as the training set and LIRI-JP as the validation set. A six-gene model calculating the risk scores of patients was integrated with the least absolute shrinkage and selection operator (LASSO) and stepwise regression analyses. Kaplan–Meier (KM) and receiver operating characteristic (ROC) curves were performed to assess predictive performances. Multivariate Cox regression analyses were implemented to select independent prognostic factors, and a prognostic nomogram was integrated. Moreover, the diagnostic values of six genes were explored with the ROC curves and immunohistochemistry.

**Results:**

Patients could be separated into two subtypes with different prognoses in both cohorts based on the identified differential hallmark pathways. Six prognostic genes (*ASF1A*, *CENPA*, *LDHA*, *PSMB2*, *SRPRB*, *UCK2*) were included in the risk score signature, which was demonstrated to be an independent prognostic factor. A nomogram including 540 patients was further integrated and well-calibrated. ROC analyses in the five cohorts and immunohistochemistry experiments in solid tissues indicated that *CENPA* and *UCK2* exhibited high and robust diagnostic values.

**Conclusions:**

Our study explored a promising prognostic nomogram and diagnostic biomarkers in hepatocellular carcinoma.

## Introduction

Hepatocellular carcinoma (HCC) is the most common liver cancer and the fourth leading cause of tumor-induced death worldwide (1). Based on recent cancer reports, mortality due to hepatocellular carcinoma has been rising rapidly compared with other cancer-related deaths in both men and women (2). Due to the insidious onset of HCC and the need for viable treatment strategies, the prognosis of HCC remains very poor, and the 5-year relative survival rate is no more than 10% (3). In this manner, there is an urgent need to recognize robust and accurate biomarkers for HCC. Multifactor models have performed extraordinary potential for future applications. In one investigation on an expansive breast cancer meta-dataset, straightforward multigene models reliably outflanked single-gene biomarkers in all segments (4). In another survey on classifiers to predict breast cancer recurrences, integrated classifiers were much better than routine biomarkers (ER, PR, HER2, Ki67) (5).

Gene set variation analysis (GSVA) is an enrichment strategy for quantifying the activities of gene sets in an unsupervised way for microarray or RNA-sequencing data (6). It has become an effective method for cancer subtype discovery or other biological issues. For instance, one investigation on the microenvironment of lung cancer conducted this algorithm to quantify the variations of relative gene sets in malignant and non-malignant cells, which led to a deeper understanding of cell subtypes and heterogeneities (7). In another investigation on the exploration of subtypes foreseeing the responses to immune checkpoint inhibitors, this method was also used to quantify the activity of a gene set controlling DNA damage and repair (8).

In this study, we quantified and differentially analyzed the activities of 50 hallmark pathways in five cohorts with GSVA (9). Patients could be separated into two subtypes with significant prognostic differences in two RNA-sequencing datasets based on the 10 identified differential hallmark pathways. Differentially expressed genes between subtypes were identified as initial variables. Then, an accurate and robust prognostic six-gene model estimating risk scores of HCC patients was constructed with the least absolute shrinkage and selection operator (LASSO) and stepwise regression analyses (10). Six prognostic genes (*ASF1A*, *CENPA*, *LDHA*, *PSMB2*, *SRPRB*, *UCK2*) were included, and the risk score was indicated to be an independent prognostic factor. Then, a well-calibrated nomogram including 540 patients was integrated (11). Receiver operating characteristic (ROC) curve analyses in the five cohorts and immunohistochemistry experiments in solid tissues indicated that *CENPA* and *UCK2* exhibited high and robust diagnostic values. In summary, our study explored a promising GSVA-based prognostic nomogram and diagnostic biomarkers in hepatocellular carcinoma.

## Materials and Methods

### Data Resources

There were five datasets selected for our work. GSE57957 (counting 39 paired tissues), GSE39791 (counting 72 paired tissues), and GSE14520 (counting 247 cancer and 241 paired adjacent tissues) were obtained from the GEO database (https://www.ncbi.nlm.nih.gov/geo). These three microarray datasets were utilized to identify differential hallmark gene sets of overlap and for diagnostic analysis. The TCGA-LIHC dataset was downloaded from The Cancer Genome Atlas (TCGA) database (https://cancergenome.nih.gov/). The LIRI-JP dataset was downloaded from the International Cancer Genome Consortium (ICGC) database (https://www.icgc.org). These two RNA-sequencing datasets were for prognostic and diagnostic analyses.

### Data Processing

The overall processing progress of this study is shown in **Supplementary Figure 1**. The expression data of the three microarray datasets were normalized with the limma R package in R 4.0.3. RNA-sequencing data of the TCGA-LIHC and LIRI-JP datasets were analyzed using fragments per kilobase per million (FPKM). Batch effect correction was removed with the SVA R package. Log_2_(*x* + 1) transformation was conducted. TCGA-LIHC (named as the TCGA cohort in this study) was used as the training cohort (*n* = 329), and LIRI-JP (designated as the ICGC cohort in this study) was used as the external validation cohort (*n* = 232). Patient characteristics in the training and the independent external validation cohorts are shown in **Table 1**.

**Table 1:** Patient characteristics in the training and the independent extra validation cohorts.

Training cohort				Extra validation cohort			
Characteristics		Number of cases	% (percentage)	Characteristics		Number of cases	% (percentage)
Age at initial diagnosis (years)		329 (16–90)		Age at initial diagnosis (years)		232 (31–89)	
Age				Age			
	>65	119	36.17		>65	142	61.21
	≤65	210	63.83		≤65	90	38.79
Gender				Gender			
	Men	226	68.69		Men	171	73.71
	Women	103	31.31		Women	61	26.29
Tumor stage				Tumor stage			
	I	155	47.11		I	36	15.52
	II	74	22.49		II	106	45.69
	III	76	23.10		III	71	30.60
	IV	3	0.91		IV	19	8.19
	Unknown	21	6.38				
Grade				Prior malignancy			
	1	50	15.20		Yes	30	12.93
	2	155	47.11		No	202	87.07
	3	107	32.52				
	4	12	3.65				
	Unknown	5	1.52				

### Identification of Differential Gene Sets

Considering the technical differences between microarray and RNA sequencing (12), we used the three microarray datasets with paired tissues to select differential gene sets. The activities of the 50 hallmark pathways were quantified with the GSVA R package, and differential analyses were performed with the limma R package. The cutoff values were selected as false discovery ratio (FDR) <0.05 and |fold change| >0.3. Common differential gene sets were identified with the Venn diagram.

### Tumor Subtypes and Differentially Expressed Genes

The overlapping differential gene sets were considered to play important roles in oncogenesis and advancement of HCC. Based on the activity profiles of differential gene sets in the TCGA and ICGC cohorts, tumor subtypes were determined with the ConsensusClusterPlus R package. Survival analyses were subsequently performed between subtypes with the survival R package. As expected, the identified hallmark gene sets contributed to different prognoses. Then, differentially expressed genes (DEGs) between subtypes were screened in the two cohorts separately. The cutoff values were selected as FDR <0.05 and absolute fold change >1. The identified differentially expressed genes were chosen as initial variables.

### Exploration of the Prognostic Signature

The LASSO and stepwise regression analyses were finally applied, and a multigene prognostic signature estimating risk scores of HCC patients was explored. Based on the median risk score value of the TCGA cohort, patients were separated into high-risk and low-risk groups. The predictive performances at 1, 2, 3, 4, and 5 years were verified using the time-dependent ROC curves. Then, the univariate and multivariate Cox regression analyses were conducted to select the independent prognostic factors. The hazard ratios (HR) and *P-*values were calculated. The area under the curve (AUC) values of the ROC curves were calculated to reveal the prediction ability with the survivalROC R package. Moreover, estimations of responses to commonly used drugs (IC50) and tumor immune dysfunction and exclusion (TIDE) scores were conducted with the pRRophetic R package (13) and TIDE webtool (http://tide.dfci.harvard.edu/) (14). Wilcoxon tests were implemented to reveal the clinical relevance between risk score and clinical characteristics.

### Integration of the Nomogram

The independent prognostic factors were integrated into a Cox model with the multivariate Cox regression analysis. A novel prognostic nomogram including 540 patients from the TCGA and ICGC cohorts was generated with the rms R package. The predictive performances at 1, 2, 3, 4, and 5 years were tested using the time-dependent ROC curves and calibration curves.

### Exploration of the Diagnostic Values and Immunohistochemistry

The diagnostic values of the identified prognostic genes within the signature in RNA expression levels were explored by using the pROC R package. The differential expressions were also confirmed in clinical tissues by immunohistochemistry. We collected paired HCC and non-tumor tissues from 30 patients treated in our hospital with the agreement of the ethics committee and obtained informed consent from each patient. The details of the clinical samples are shown in **Table 2**. The experiments were performed according to the Helsinki Declaration. Primary antibodies (rabbit anti-CENPA, 1:100, Invitrogen, Carlsbad, USA, MA1-20832; rabbit anti-UCK2, 1:100, Invitrogen, Carlsbad, USA, PAS-14010) were used for staining. Images of each section were obtained at magnifications of ×100 and ×400. The mean integral optical density (IOD/Area) values were used for the quantitative analyses with Image-Pro Plus 6.0. Differential analyses were performed in GraphPad Prism 8 software with the paired *t*-test. The ROC analyses were further performed to explore the diagnostic efficacy in the protein levels with the pROC R package.

**Table 2:** Clinicopathological characteristics.

Characteristics		Number of cases	% (percentage)
Age			
	≥65	4	13.33
	<65	26	86.67
Gender	Men	19	63.33
	Women	11	36.67
Tumor stage			
	I	2	6.67
	II	23	76.67
	III	5	16.67
Grade			
	1	1	3.33
	2	20	66.67
	3	9	30.00

## Results

### Identification of the Differential Gene Sets

**Figure 1:**
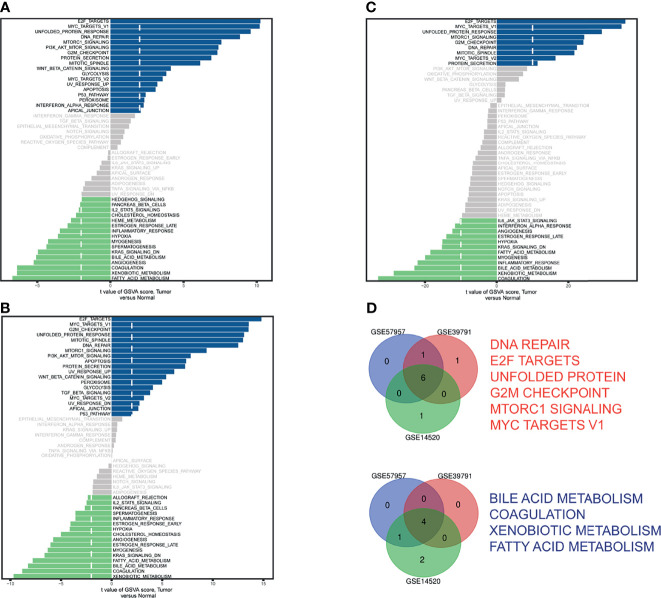
Identification of differential hallmark pathways in **(A)** GSE57957, **(B)** GSE39791, and **(C)** GSE14520. **(D)** Venn diagram of upregulated pathways (red) and downregulated pathways (blue).

The GSVA scores of 50 hallmark pathways in GSE57957 (**Figure 1A**), GSE39791 (**Figure 1B**), and GSE14520 (**Figure 1C**) were visualized in bar plots. The Venn diagram identified six upregulated and four downregulated (**Figure 1D**) pathways. The GSVA scores and differential analysis results were provided (**Supplementary Table 1**).

### Tumor Subtypes and Differentially Expressed Genes

**Figure 2:**
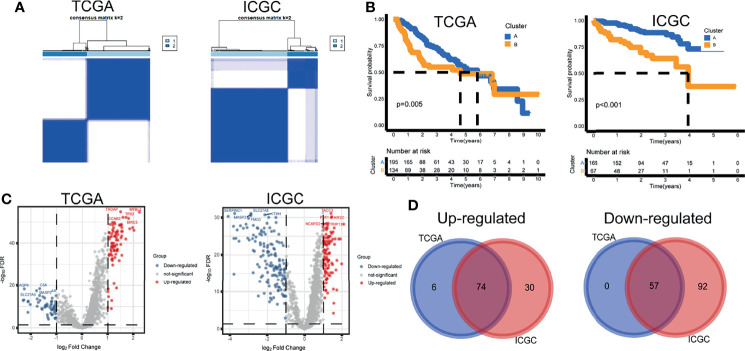
Tumor subtypes and differentially expressed genes. **(A)** Unsupervised clustering. **(B)** Kaplan–Meier curves. **(C)** Volcano maps of differentially expressed genes. **(D)** Venn diagram of upregulated and downregulated genes.

Based on the activity profiles of the 10 differential gene sets, patients could be grouped into cluster A and cluster B in the TCGA and ICGC cohorts (**Figure 2A**). Interestingly, significant differences in prognosis were found in both cohorts (**Figure 2B**). Differential analyses of 1,368 background genes within the differential gene sets were conducted between cluster B and cluster A. The volcano maps of DEGs were plotted (**Figure 2C**). Seventy-four upregulated and 57 downregulated DEGs were screened by the Venn diagram (**Figure 2D**). These survival-related DEGs were selected as the initial variables for the following LASSO regression analysis. The GSVA scores, clustering information, and differential analysis results were provided (**Supplementary Table 2**).

### Exploration of the Prognostic Signature

**Figure 3:**
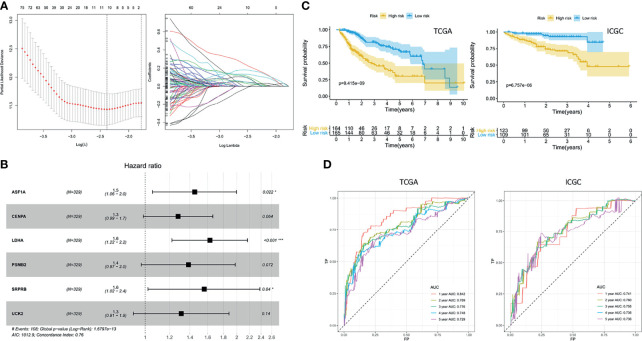
Exploration of the prognostic signature. **(A)** Least absolute shrinkage and selection operator analysis. Determination of lambda (left); variations of coefficients (right). **(B)** Hazard ratios. **(C)** Kaplan–Meier curves. **(D)** Receiver operating characteristic curve (ROC) curves.

**Figure 4:**
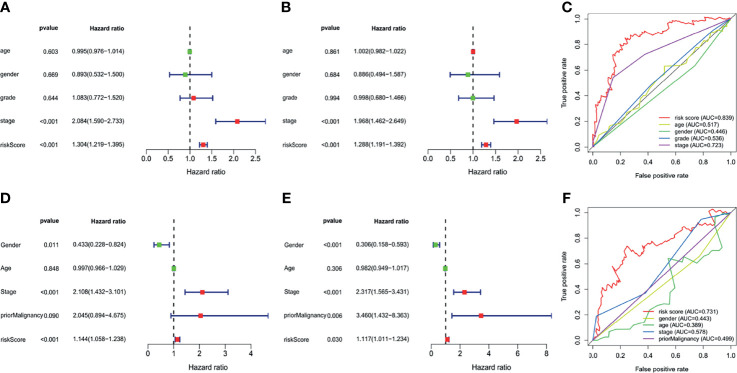
Independent prognostic analyses. **(A)** Univariate analysis of The Cancer Genome Atlas (TCGA). **(B)** Multivariate analysis of the TCGA. **(C)** Multifeature ROC curve in the TCGA. **(D)** Univariate analysis of the International Cancer Genome Consortium (ICGC). **(E)** Multivariate analysis of the ICGC. **(F)** Multifeature ROC curve in the ICGC.

**Figure 5:**
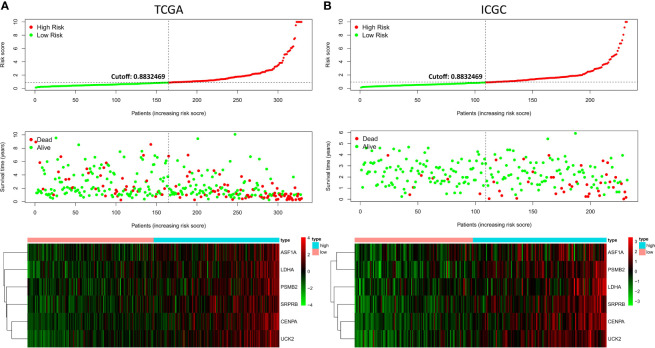
Risk factor correlation curve. **(A)** Risk scores, survival status, and gene expressions in the TCGA. **(B)** Risk scores, survival status, and gene expressions in the ICGC.

**Figure 6:**
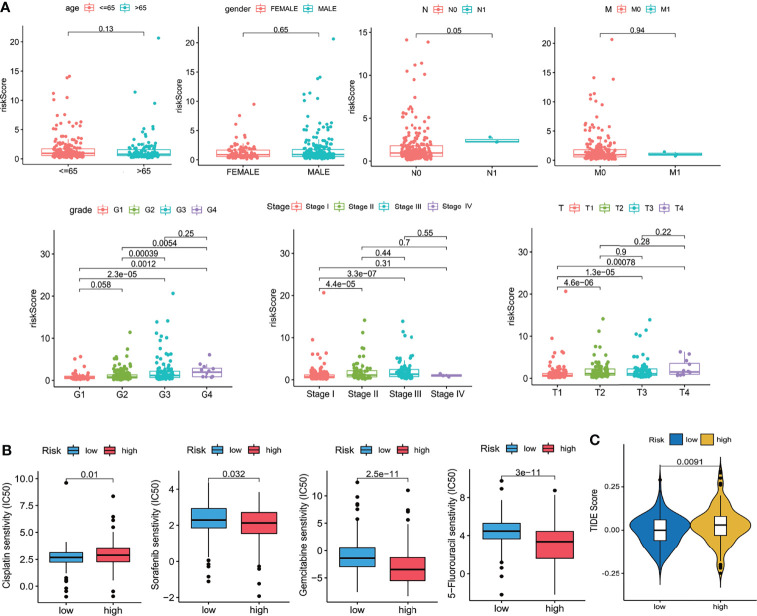
Clinical relevance. **(A)** Clinical relevance with age, gender, grade, and TNM stages. **(B)** Drug sensitivity scores (IC50 estimations). **(C)** TIDE scores. Wilcoxon test.

Ten prognostic genes were identified with the LASSO regression analysis in the TCGA cohort (**Figure 3A**), and the expression profile with survival data was provided (**Supplementary Table 3**). Then, a six-gene signature was finally explored with the concordance index = 0.76 (**Figure 3B**). The formula calculating risk scores was as follows: risk score = (0.372283552 × expression level of *ASF1A*) + (0.247238902 × expression level of *CENPA*) + (0.487191218 × expression level of *LDHA*) + (0.325756023 × expression level of *PSMB2*) + (0.443769932 × expression level of *SRPRB*) + (0.270784173 × expression level of *UCK2*). Based on the median risk score of 0.8832469 in the TCGA cohort, patients were separated into high-risk and low-risk groups, and the risk scores of all patients in the two cohorts were provided (**Supplementary Table 3**). The high-risk group had a significantly worse prognosis in both cohorts (*P* < 0.001) (**Figure 3C**). The AUC values of the ROC curves were 0.842, 0.789, 0.776, 0.748, and 0.729 in the TCGA cohort and 0.741, 0.760, 0.755, 0.736, and 0.736 in the ICGC cohort from 1 to 5 years (**Figure 3D**). Then, the univariate and multivariate Cox regression analyses were conducted to select the independent prognostic factors. There were 214 patients with integral clinical data from the TCGA cohort, and 228 patients from the ICGC cohort were included (**Supplementary Table 4**). According to the results of the univariate and multivariate Cox regression analyses, risk score and stage were the two independent variables with *P < *0.05 in the TCGA (**Figures 4A, B**) and ICGC (**Figures 4D, E**) cohorts. Moreover, risk score showed the highest AUC value of 0.839 and 0.731 within the 5-year ROC curves of the TCGA (**Figure 4C**) and ICGC (**Figure 4F**) cohorts. The distributions of gene expression levels and survival data along with increasing risk were visualized in the TCGA (**Figure 5A**) and ICGC (**Figure 5B**) cohorts. Risk score was not associated with age, gender, or metastasis (*P* > 0.05). However, group N1 had higher risk scores than group N0 (*P* < 0.05), and group G4 and group G3 had higher risk scores than group G1 and group G2 (*P* < 0.01). Patients in group T1 had lower risk scores than those in groups T2, T3, and T4. Patients in stage I showed lower risk scores than those in stages II and III (*P* < 0.001) (**Figure 6A**). Drug sensitivity estimation results showed that the low-risk group was more sensitive to cisplatin (*P* = 0.01) and less sensitive to sorafenib (*P* = 0.032), gemcitabine (*P* < 0.001), and 5-fluorouracil (*P* < 0.001) (**Figure 6B**). However, the high-risk group had significantly (*P* = 0.0091) higher TIDE scores (**Figure 6C**, **Supplementary Table 5**).

### Integration of the Nomogram

**Figure 7:**
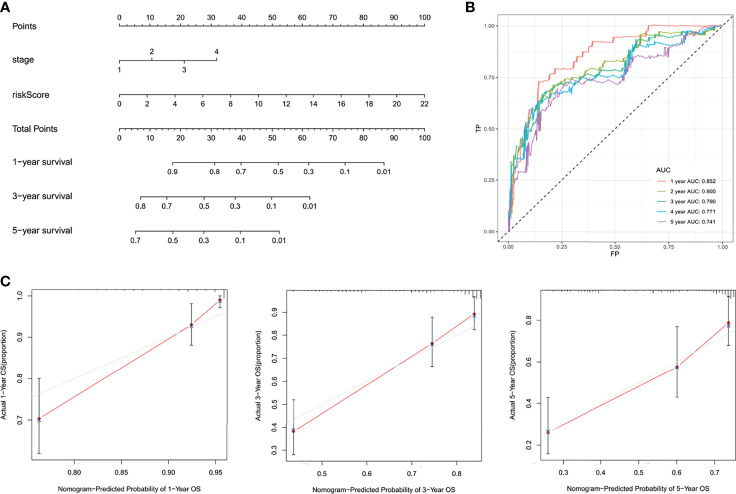
Integration of the nomogram. **(A)** Nomogram display. **(B)** The ROC curves. **(C)** The calibration curves.

The independent factors (stage and risk score) were integrated into a novel prognostic nomogram by the multivariate Cox regression analysis (**Figure 7A**). The AUC values of the ROC curves were all over 0.7 from 1 to 5 years (**Figure 7B**), and the integrated nomogram was well-calibrated (**Figure 7C**).

### Exploration of the Diagnostic Values and Immunohistochemistry

**Figure 8:**
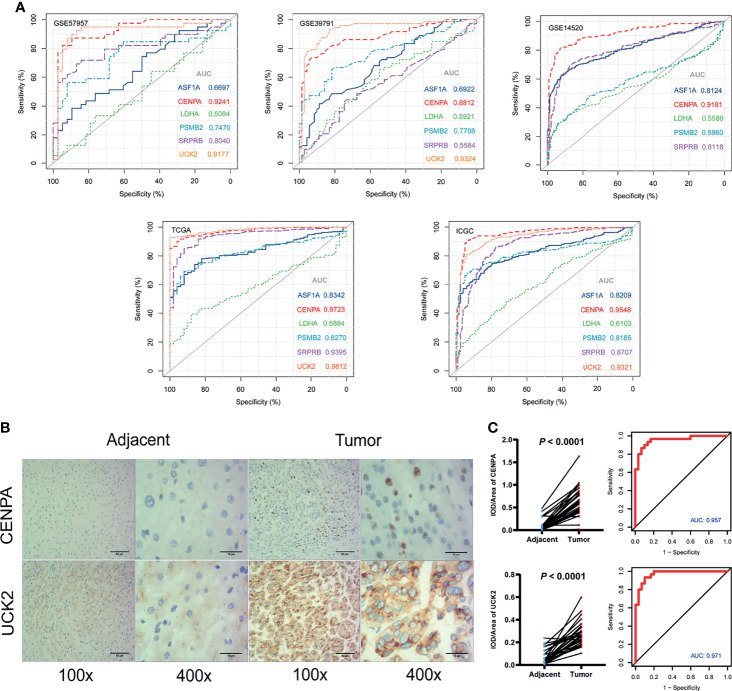
Diagnostic values of the identified prognostic genes. **(A)** The diagnostic ability assessment was based on the area under the curve (AUC) values. AUC over 0.9 means high predictive ability and AUC from 0.7 to 0.9 means medium predictive ability. Note: *UCK2* was not available in GSE14520. **(B)** Immunohistochemistry. Representative images of *CENPA* and *UCK2* in tumor and adjacent non-tumor tissues. Brown color means positive staining areas. Length of the scale bars are 50 and 15 µm. **(C)** Statistical results. (Left) The mean integral optical density (IOD/Area) values and statistical results. Paired *t*-test. (Right) The diagnostic ROC curves of *CENPA* and *UCK2*.

In the five cohorts included in our study, the AUC values of *CENPA* in the RNA expression levels were 0.92 (GSE57957), 0.88 (GSE39791), 0.92 (GSE14520), 0.97 (TCGA), and 0.95 (ICGC) (**Figure 6A**). The AUC values of *UCK2* in the RNA expression levels were 0.92 (GSE57957), 0.93 (GSE39791), 0.98 (TCGA), and 0.93 (ICGC) separately (**Figure 8A**, **Supplementary Table 6**). Among the six genes, *CENPA* and *UCK2* exhibited more robust and better predictive performances, which was verified in the immunohistochemistry experiment. Representative pictures of tumor and adjacent non-tumor sections were shown, and brown indicated positive immunohistochemical staining (**Figure 8B**). The mean integral optical density (IOD/Area) values of *CENPA* and *UCK2* (**Supplementary Table S7**) were significantly upregulated in the tumor tissues (*P* < 0.0001) (**Figure 8C**). The AUC values of the ROC curves in protein levels reached 0.957 for *CENPA* and 0.971 for *UCK2* (**Figure 8C**).

## Discussion

Alterations of signaling pathways play a pivotal role in tumorigenesis and cancer progression (15). With advantages in genome-sequencing innovation, important molecular pathways were identified to be responsible for the occurrence and progress of HCC (16). Critical pathways, for instance, RAF/MEK/ERK, PI3K/AKT/mTOR, WNT/β-catenin, HGF/c-MET, and angiogenesis pathways, have been found, and relative treatments have been investigated (17). Despite the advantages of small-molecule targeted therapy and immunotherapy, the survival of HCC patients is far from ideal (18, 19). Conventional biomarkers like AFP and TNM stages show limited predictive ability (20). Utilizing the strategy of mathematical and statistical modeling, prediction models based on gene expression profiles have incredible application potential (21). To date, many efforts have been made to reach this point. For example, the prognostic roles of N6-methyladenosine (m6A)-related genes in the TCGA cohort were discovered (22). Hypoxia-related genes and the relative signature were explored to predict survival in HCC (23). Immune-related and ferroptosis-related signatures were also conducted by researchers (24–26). However, all of these studies focused on one specific function or pathway initially. Whether they play critical roles in the prognosis of cancer or a specific cancer type is a concern that needs further study. In addition, there were complex cross talks or interactions among different pathways working together to influence the occurrence and development of HCC (27, 28).

Our study quantified and differentially analyzed the activities of 50 hallmark pathways in five cohorts with GSVA. Based on the activities of the 10 identified differential hallmark pathways, patients could be separated into two subtypes with significant prognostic differences. Differentially expressed genes between subtypes were identified as the initial variables associated with overall survival. Then, an accurate and robust prognostic six-gene model estimating the risk scores of HCC patients was constructed with the LASSO and stepwise regression analyses. Six prognostic genes (*ASF1A*, *CENPA*, *LDHA*, *PSMB2*, *SRPRB*, *UCK2*) were included, and risk score was indicated to be an independent prognostic factor for the prognosis of HCC. In addition, ROC analyses in the five cohorts and immunohistochemistry experiments in solid tissues indicated that *CENPA* and *UCK2* exhibited high and robust diagnostic values. All the six genes were unfavorable factors with hazard ratios over 1.2 in the Cox model. Patients with higher risk scores reflected worse clinical phenotypes, especially higher pathological grades and TNM stages. Also, higher TIDE scores demonstrated that patients in the high-risk group might be restricted with more serious immune evasion of cancer cells (29). The drug sensitivity (IC50 scores) results in our study indicated that patients in the low-risk group were more sensitive to cisplatin and less sensitive to sorafenib, gemcitabine, and 5-fluorouracil. This phenomenon suggests that immunotherapy might be more suitable for low-risk patients and chemotherapy might be more suitable for high-risk patients. However, it is known that the majority of HCC cells were tolerant to chemotherapy drugs (30), so further explorations in the drug responses are needed. Nomograms are gradually utilized as predictive tools for clinicians (31). For clinical practices, the independent factors (stage and risk score) were integrated into a prognostic nomogram including up to 540 patients by multivariate Cox regression analysis in our study.

Besides prognostic values, *CENPA* and *UCK2* also exhibited robust and accurate diagnostic values in our study. The AUC values of the two genes in RNA expression levels reached over 0.9 in four of the five independent cohorts and reached over 0.95 in protein levels in the immunohistochemistry validation cohort. The role of *CENPA* in some cancer types like prostate cancer (32), colon cancer (33), breast cancer (34), gastric cancer (35), and head and neck cancer (36) was widely reported. Although the carcinogenicity of *CENPA* in HCC has been explored by a few bioinformatic analyses (37, 38), the *CENPA*-mediated molecular mechanisms in HCC remain not so clear. Particularly, the diagnostic value of *CENPA* in HCC was also not well explored. As for *UCK2*, it was reported to be related to unfavorable prognosis and metastasis in HCC (39, 40). *In-vitro* and *in-vivo* experiments also proved the high association with HCC malignant behavior (41). However, the role of *UCK2* in the diagnosis of HCC was not fully studied. These pieces of evidence jointly indicated the potential application values of *CENPA* and *UCK2* in the diagnosis and prognosis of HCC.

For better clinical applications, we transformed the prognostic nomogram into a web tool with Shiny (https://shiny.rstudio.com/). In brief, clinicians can calculate the risk score of each patient with the previously mentioned formula based on the gene expressions of the six prognostic genes. Then, the risk score and stage can be entered into the web tool (https://survival-prediction.shinyapps.io/prognostic-nomogram-hcc/), and the survival predictions can be easily performed. On the other hand, the mRNA or protein expression levels of *CENPA* or *UCK2* can also be applied in the early diagnosis of HCC.

This study has some limitations. First, there were merely two datasets included based on next-generation sequencing technology to integrate the prognostic nomogram within 540 patients in our study. Larger samples and more independent cohorts based on the same sequencing technique are expected to validate predictability. Second, this study did not provide insight into in-depth mechanisms, which we will make as the focus in our future studies.

## Conclusions

In this study, six prognostic genes (*ASF1A*, *CENPA*, *LDHA*, *PSMB2*, *SRPRB*, *UCK2*) were identified, and a novel six-gene signature was constructed to predict the prognosis of HCC patients. The signature and clinical features were further integrated into a well-calibrated nomogram which showed an accurate and robust performance. In addition, ROC analyses in the five cohorts and immunohistochemistry experiments in solid tissues indicated that *CENPA* and *UCK2* exhibited high and robust diagnostic values.

## Data Availability Statement

The raw data supporting the conclusions of this article will be made available by the authors, without undue reservation.

## Ethics Statement

The studies involving human participants were reviewed and approved by the Ethics Committee of Zhuhai People’s Hospital. The patients/participants provided their written informed consent to participate in this study.

## Author Contributions

XZ and HC designed the study. XZ, HC, and XY collected and analyzed the data. XZ and HC wrote the article. All authors contributed to the article and approved the submitted version.

## Funding

This study was supported by Guangdong Basic and Applied Basic Research Foundation (2019A1515011763).

## Conflict of Interest

The authors declare that the research was conducted in the absence of any commercial or financial relationships that could be construed as a potential conflict of interest.

## Publisher’s Note

All claims expressed in this article are solely those of the authors and do not necessarily represent those of their affiliated organizations, or those of the publisher, the editors and the reviewers. Any product that may be evaluated in this article, or claim that may be made by its manufacturer, is not guaranteed or endorsed by the publisher.

## Supplementary Material

Supplementary Figure 1 The design and data processing progress of this study.

Supplementary Table 1 The GSVA scores and differentially analysis results of GSE57957, GSE39791 and GSE14520.

Supplementary Table 2 The GSVA scores, clustering information and differentially analysis results of the TCGA and ICGC cohorts.

Supplementary Table 3 The risk scores of all the patients in the TCGA and ICGC cohorts.

Supplementary Table 4 The clinical data for the independent prognostic analyses.

Supplementary Table 5 The tumor immune dysfunction and exclusion (TIDE) scores.

Supplementary Table 6 The expression profiles of the identified genes in the five cohorts.

Supplementary Table 7 The mean integral optical density (IOD/Area) values of CENPA and UCK2.

## Abbreviations

GSVA, gene set variation analysis; DEGs, differentially expressed genes; FPKM, fragments per kilobase per million; FDR, false discovery ratio; HR, hazard ratio; ROC, receiver operating characteristic curve; AUC, area under the curve; TIDE, tumor immune dysfunction and exclusion.
