# Supplementary figures and images for: Exploration of a Novel Prognostic Nomogram and Diagnostic Biomarkers Based on the Activity Variations of Hallmark Gene Sets in Hepatocellular Carcinoma
Source: Front Oncol. 2022 Mar 10;12:830362. doi: 10.3389/fonc.2022.830362 (PMC8960170; doi:10.3389/fonc.2022.830362)

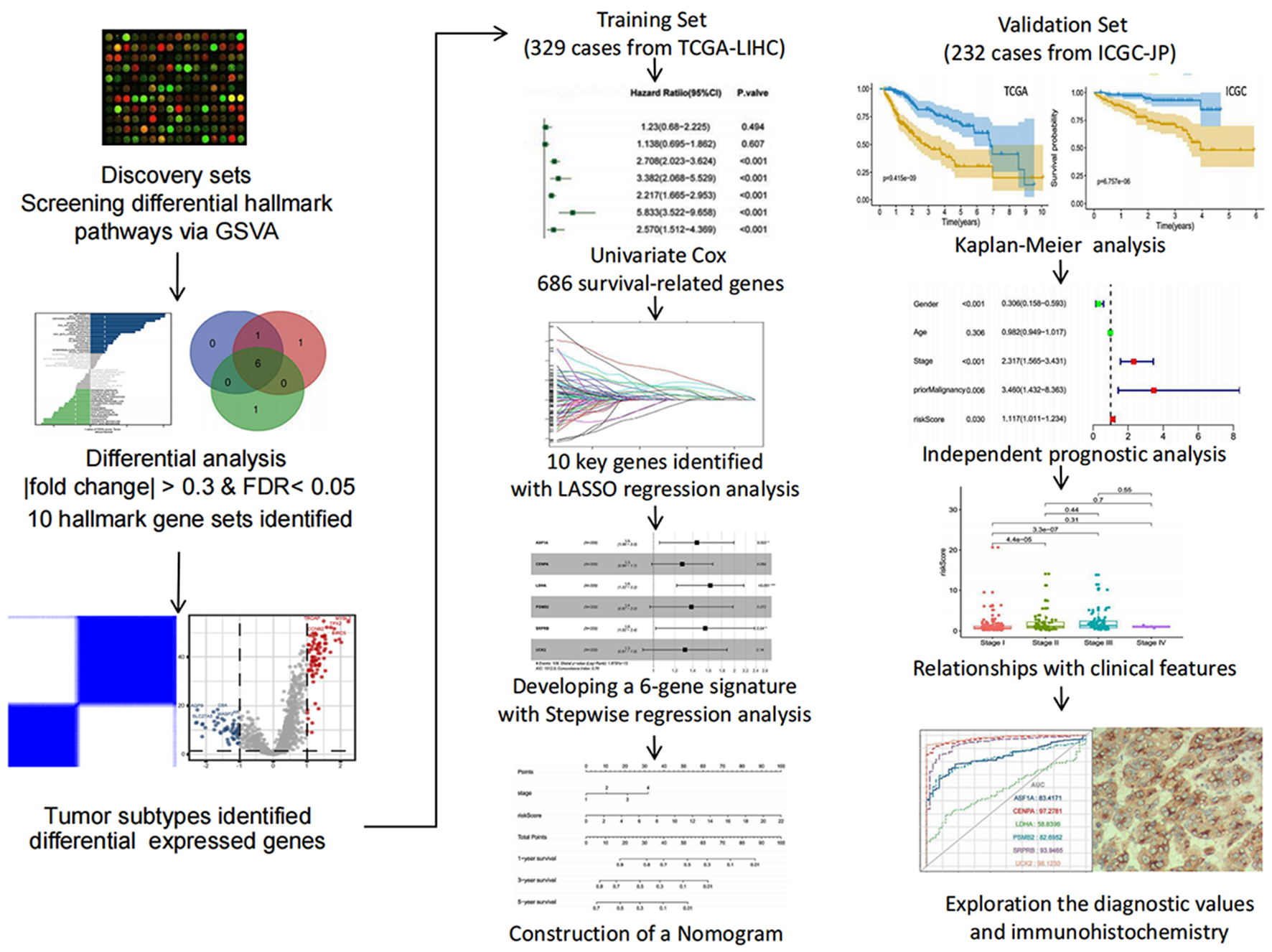

Supplement: Supplementary Figure 1 — The design and data processing progress of this study. [file Image_1.tif]
